# Large-scale high purity and brightness structural color generation in layered thin film structures via coupled cavity resonance

**DOI:** 10.1515/nanoph-2024-0471

**Published:** 2024-10-30

**Authors:** Danyan Wang, Chengang Ji, Moxin Li, Zhenyu Xing, Hao Gao, Xiaochan Li, Huixian Zhou, Yuhui Hu, Zhelin Lin, Cheng Zhang

**Affiliations:** School of Optical and Electronic Information & Wuhan National Laboratory for Optoelectronics, 12443 Huazhong University of Science and Technology, Wuhan, Hubei 430074, China; Ningbo Inlight Technology Co., Ltd, Ningbo, Zhejiang 315500, China

**Keywords:** structural color, Fabry-Pérot cavity, layered thin film structure, coupled cavity resonance

## Abstract

Structural colors, resulting from the interaction of light with nanostructured materials rather than pigments, present a promising avenue for diverse applications ranging from ink-free printing to optical anti-counterfeiting. Achieving structural colors with high purity and brightness over large areas and at low costs is beneficial for many practical applications, but still remains a challenge for current designs. Here, we introduce a novel approach to realizing large-scale structural colors in layered thin film structures that are characterized by both high brightness and purity. Unlike conventional designs relying on single Fabry–Pérot cavity resonance, our method leverages coupled resonance between adjacent cavities to achieve sharp and intense transmission peaks with significantly suppressed sideband intensity. We demonstrate this approach by designing and experimentally validating transmission-type red, green, and blue colors using an Ag/SiO_2_/Ag/SiO_2_/Ag configuration on fused silica substrate. The measured spectra exhibit narrow resonant linewidths (full width at half maximum ∼60 nm), high peak efficiencies (>40 %), and well-suppressed sideband intensities (∼0 %). In addition, the generated color can be easily tuned by adjusting the thickness of SiO_2_ layer, and the associated color gamut coverage shows a wider range than many existing standards. Moreover, the proposed design method is versatile and compatible with various choices of dielectric and metallic layers. For instance, we demonstrate the production of angle-robust structural colors by utilizing high-index Ta_2_O_5_ as the dielectric layer. Finally, we showcase a series of printed color images based on the proposed structures. The coupled-cavity-resonance architecture presented here successfully mitigates the trade-off between color brightness and purity in conventional layered thin film structures and provides a novel and cost-effective route towards the realization of large-scale and high-performance structural colors.

## 1 Introduction

Structural colors, arising from the resonant interaction between light and nanostructures, exhibit several unique advantages compared to their conventional counterparts based on pigments or dyes, such as long-term stability, fine spatial resolution, and multiplexed functionality [1], [2], [3], [4], [5], [6]. Structural colors have gained increasing interest due to their potential applications in various fields including ink-free printing [7], [8], [9], optical sensing [10], [11], energy harvesting [12], [13], and optical anti-counterfeiting [14], [15]. Structural colors can be generated from either arrayed sub-wavelength nanostructures [16] or layered thin film structures [17], [18]. Different resonance effects, such as guided mode resonance [19], [20], [21], plasmon resonance [22], [23], [24], and Mie resonance [25], [26], [27], can be engineered to take place in arrayed sub-wavelength nanostructures and create colors with fine spatial resolution, high efficiency, wide gamut coverage, and multiplexed functionality. However, the fabrication of sub-wavelength nanostructure-based colors typically involves a series of high-resolution lithography and well-controlled pattern transfer processes which are often time-consuming and costly, thereby limiting the devices’ feasibility for large-scale and low-cost applications. In contrast, layered thin film structures present a practical alternative for realizing high-performance structural colors over a large scale and in a cost-effective manner [28], [29], [30], [31]. One representative structure of such kind is based on the Fabry–Pérot (FP) cavity, typically composed of an optically transparent dielectric medium sandwiched between two metallic mirrors. By adjusting the material or thickness of the dielectric (metallic) layer to manipulate the resonance effect supported by the FP cavity, a range of reflection- and transmission-type colors can be easily realized.

For various applications, there is a considerable demand for structural colors that simultaneously exhibit high purity and brightness. However, due to the intrinsic tradeoff between the above two performance parameters and the limited design freedom of typical FP cavity structures, achieving colors with both high purity and brightness is often challenging. The fundamental resonances, which are commonly utilized for color generation in FP cavities, typically yield transmission (reflection) spectra with relatively broad linewidths and high sideband intensities, rendering low purity of the generated colors. To mitigate such limitation, high-order resonances which are characterized by narrower linewidth compared to the fundamental resonance and created by increasing the FP cavity length, are proposed for high-purity color generation [32], [33]. Such designs, however, will unavoidably introduce undesired resonances at shorter wavelengths. To suppress these undesired resonances, one or more thin absorbing layers need to be strategically added into the FP cavities [34]. Nevertheless, the addition of absorbing layers compromises the intensity of resonant peaks, resulting in reduced color brightness. Another proposed approach for enhancing color purity involves the suppression of undesired sideband intensity through the use of highly absorbing materials. High-extinction-coefficient metals such as nickel (Ni) [35] and tungsten (W) [36], or semiconductors such as amorphous silicon (α-Si) [37] and germanium (Ge) [36], [38], have been employed in constructing FP cavities which produce high purity structural colors with largely-suppressed sideband intensities. However, achieving a specific color in these structures requires the utilization of one or more absorbing layers with particular refractive indices and thicknesses, making the design and fabrication process relatively intricate. Furthermore, similar to the first strategy, these absorbing layers unavoidably affect the intensity of resonant peaks, compromising the colors’ brightness. A third approach involves the use of dual-cavity structures, where different combinations of dielectric and metallic layer thicknesses are adjusted for each target color (red, green, and blue) [39]. Despite these adjustments, the spectral purity and intensity produced by these structures are often inadequate. To further improve the device performance, anti-reflection coatings are applied on both the top and bottom surfaces, with the thickness of each coating individually optimized for every intended color.

In this study, we present a new approach for creating large-scale structural colors characterized by both high brightness and purity, utilizing a metal/dielectric/metal/dielectric/metal (MDMDM) configuration. Departing from conventional methods reliant on the resonance effect within a single Fabry–Pérot (FP) cavity, our proposed structure capitalizes on the coupled resonance between two adjacent FP cavities. Such design yields a sharp and intense transmission peak from the layered structures accompanied by a significantly suppressed sideband intensity, thereby resulting in colors of both high brightness and purity. We validate the practicality of our approach by designing and fabricating transmission-type red (R), green (G), and blue (B) colors using an Ag/SiO_2_/Ag/SiO_2_/Ag configuration on fused silica substrate. The measured transmission spectra of these RGB devices exhibit narrow resonant linewidths (with a full width at half maximum, FWHM, of approximately 60 nm) and high peak efficiencies exceeding 40 %, with sideband intensities effectively minimized to around 0 %. Furthermore, by adjusting the thickness of the SiO_2_ layers, we demonstrate the ability to produce colors spanning the entire visible spectrum, resulting in a wide color gamut that occupies 195.0 % of sRGB, 144.5 % of Adobe RGB, and 102.7 % of Rec. 2020 in the CIE 1931 XYZ chromaticity diagram. Additionally, our MDMDM structure exhibits high-performance complementary colors (cyan, magenta, and yellow) in reflection view, further expanding its versatility. Notably, the proposed MDMDM architecture is compatible with various choices of metallic and dielectric layers. For instance, we illustrate the production of angle-robust structural colors by utilizing high-index Ta_2_O_5_ as the dielectric layer. In the end, we showcase a group of printed color images based on the proposed configurations. The demonstrated MDMDM coupled-cavity-resonance architecture, with its remarkable color-generating performance and compatibility with large-scale manufacturing process, holds promise for diverse applications ranging from advanced printing and decoration to optical anti-counterfeiting and colored photovoltaics.

## 2 Results and discussion

**Figure 1:**
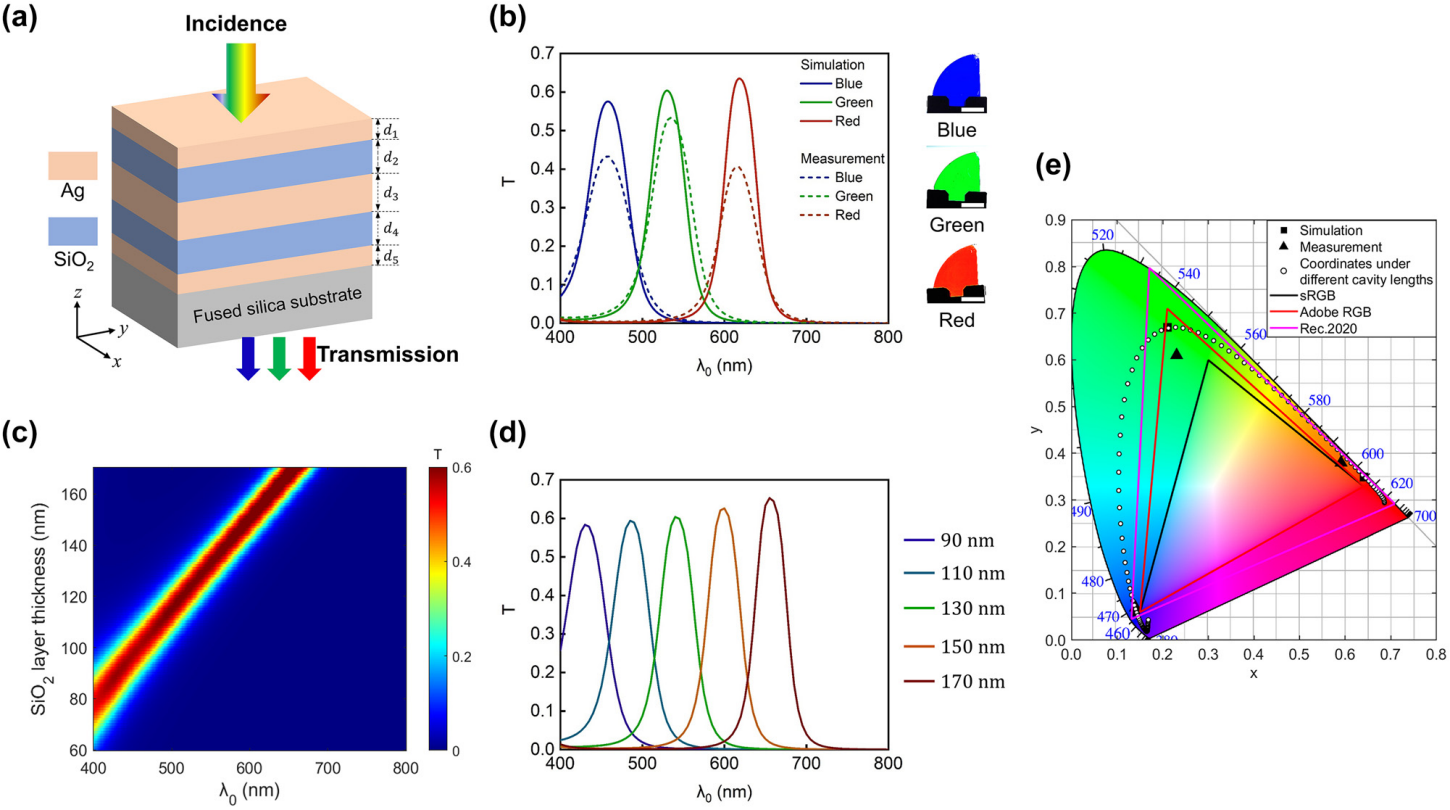
Transmission-type structural color generation based on the Ag-SiO_2_-Ag-SiO_2_-Ag configuration. (a) Schematic representation of the transmission-mode structural color generation based on an Ag–SiO_2_–Ag–SiO_2_–Ag configuration under normally-incident white light illumination. Layer thicknesses from top to bottom are represented by *d*_1_ ∼ *d*_5_. (b) Left panel: simulated (solid curves) and measured (dashed curves) transmission spectra for blue (B), green (G), and red (R) devices. Right panel: optical images (transmission-view) of the fabricated samples. Scale bars: 1.0 cm. The associated structural parameters of these devices are listed in Table 1. (c) Contour plot of the simulated transmission spectrum as functions of the SiO_2_ thickness and illumination wavelength. Here, *d*_1_ = *d*_5_ = 20 nm and *d*_3_ = 50 nm. (d) Five representative transmission spectra with SiO_2_ layer thicknesses ranging from 90 nm to 170 nm in 20 nm increments. (e) Simulated (red (0.64, 0.35), green (0.21, 0.67), blue (0.14, 0.06)) and measured (red (0.62, 0.37), green (0.25, 0.63), blue (0.14, 0.08)) color coordinates of the RGB devices, as well as three popular color gamut standards (sRGB, Adobe RGB, and Rec. 2020) in the CIE 1931 XYZ chromaticity diagram. The white points correspond to color coordinates calculated based on the simulated transmission spectra of the proposed device with SiO_2_ cavity length ranging from 60 nm to 170 nm with an increasement of 1 nm.

Figure 1a depicts the schematic illustration of the proposed device, featuring a metal-dielectric-metal-dielectric-metal (MDMDM) configuration on a fused silica substrate. In this illustration, *d*_1_ ∼ *d*_5_ respectively represents the layer thickness from top to bottom. The MDMDM configuration can be conceptualized as two closely-spaced individual MDM Fabry-Pérot (FP) cavities, whose metallic layers have half of the middle layer thickness (*d*_3_) in the MDMDM structure. Each individual FP cavity adheres to the classic three-layer configuration, comprising an optically transparent dielectric medium sandwiched between two metallic mirrors. In our study, silver (Ag) is chosen as the metallic layer for its low absorption and high reflection across the visible spectrum, while silicon dioxide (SiO_2_) serves as the middle dielectric layer by its high transparency over the visible spectrum. Figure S1 in the Supplementary Information displays the experimentally measured refractive indices (*n*) and extinction coefficients (*κ*) of Ag and SiO_2_ films, obtained using a spectroscopic ellipsometer over the wavelength range from 400 to 800 nm.

Compared to an individual MDM structure, the proposed MDMDM structure enhances purity of the generated color by significantly narrowing the resonant linewidth and simultaneously suppressing the sideband intensity of its transmission spectrum. Furthermore, the high brightness of the generated color is achieved through the device’s high peak transmission efficiency. The solid curves in Figure 1b depict the simulated transmission spectra under normal light illumination for three representative devices (the associated structural parameters are listed in Table 1), respectively exhibiting peak transmission wavelengths of 620 nm (corresponding to red color generation), 530 nm (green color generation), and 460 nm (blue color generation). In the plot, the employed color of each curve is deliberately chosen based on the standard RGB (sRGB) values calculated from the associated spectrum. These devices exhibit peak transmission intensities exceeding 55 %, with resonant linewidths of approximately 50 nm (full width at half maximum, FWHM) and suppressed sideband transmission efficiencies down to around 0 %. The large peak transmission intensity contributes to the high brightness of the generated color, and at the same time, the suppressed sideband transmission and narrowed resonant linewidth contribute to the enhanced color purity.

**Table 1:** Geometric parameters of the MDMDM devices implemented in the study. The devices can simultaneously generate transmission-view additive colors (red, green, and blue) and reflection-view subtractive colors (cyan, magenta, and yellow).

Transmission-color (peak wavelength)	Reflection-color (dip wavelength)	*d*_1_	*d*_2_	*d*_3_	*d*_4_	*d*_5_
Red (620 nm)	Cyan (630 nm)	20 nm	157 nm	50 nm	157 nm	20 nm
Green (530 nm)	Magenta (535 nm)	20 nm	126 nm	50 nm	126 nm	20 nm
Blue (460 nm)	Yellow (470 nm)	20 nm	100 nm	50 nm	100 nm	20 nm

The devices are fabricated by sequentially depositing Ag and SiO_2_ layers with designed thicknesses onto fused silica substrate (details elaborated in the Methods section). The measured transmittance of the fused silica substrate is plotted in Figure S2 of the Supplementary Information. The dashed curves in Figure 1b depict the measured transmission spectra under normal incidence, showcasing narrow resonant linewidths (∼60 nm), high peak efficiencies (>40 %), and well-suppressed sideband intensities (∼0 %), all of which exhibit reasonable correspondence with the simulated ones. Similarly, the color of each curve is chosen based on the sRGB values calculated from the corresponding measured transmission spectrum. There is certain discrepancy between the measured and simulated curves, and this could be due to several factors including surface roughness of the deposited Ag film, variation in the film thicknesses and refractive indices during the fabrication process, as well as interfacial diffusion between neighboring layers [40], [41], [42]. The simulated and measured transmission spectra of the devices under different angles of incidence and states of polarization are displayed in Section III, Supplementary Information. Optical images (transmission-view) of the fabricated blue (B), green (G), and red (R) color samples are displayed in the right panel of Figure 1b. The color coordinates, calculated based on both simulated and measured transmission spectra at normal incidence, are plotted in the CIE 1931 XYZ chromaticity diagram (Figure 1e). Simulated and measured color coordinates for each color closely align. Moreover, it is worth noting that all coordinates are positioned near the edge of the chromaticity diagram and outside the region of the standard RGB (sRGB) color gamut (area enclosed by the black line) widely used in liquid crystal displays, indicating the high purity of the colors generated by such MDMDM configuration. Details of the CIE 1931 coordinate calculation and how to analyze the CIE diagram in relation to color generation performance are elaborated in Section IV, Supplementary Information.

The peak transmission wavelength and the corresponding generated color can be easily adjusted by varying the device’s cavity length. Figure 1c displays the contour plot of the device’s simulated transmission spectrum as functions of the SiO_2_ cavity thickness and illumination wavelength. In the simulation, the thicknesses of the top and bottom Ag layers are fixed at 20 nm (*d*_1_ = *d*_5_ = 20 nm) and the middle Ag layer at 50 nm (*d*_3_ = 50 nm), while the thicknesses of the two SiO_2_ layers are set to be the same (*d*_2_ = *d*_4_) and vary from 60 nm to 170 nm. The device’s peak transmission wavelength continuously shifts from approximately 400 nm to 655 nm as the SiO_2_ layer thickness varies from 60 nm to 170 nm. With every 1 nm change in the SiO_2_ layer thickness, the peak wavelength of the transmission spectrum exhibits a shift of approximate 2.5 nm. Figure 1d presents five representative transmission spectra, with SiO_2_ layer thicknesses ranging from 90 nm to 170 nm with an increment of 20 nm. The employed color of each curve is also chosen based on the sRGB values of the corresponding spectrum. All these spectra exhibit high peak efficiencies over 55 %, a narrow resonant linewidth with FWHM of approximately 50 nm, and significantly suppressed sideband transmission close to 0 %. White points in Figure 1e are the color coordinates, each of which corresponds to a device design with a distinct SiO_2_ cavity length. As the cavity length increases, the associated coordinate point moves clockwise from the lower-left corner to the upper-left corner and then to the lower-right corner in the chromaticity diagram, and the generated color changes accordingly from blue to green and finally to red. All these color coordinates are located near the diagram’s edge, demonstrating high color purity. It is worthwhile to note that the color gamut enclosed by the white circles occupies 195.0 % of sRGB, 144.5 % of Adobe RGB, and 102.7 % of Rec. 2020, wider than recent reports based on sub-wavelength nanostructure arrays [43], [44] and layered thin films [34], [36], [45].

**Figure 2:**
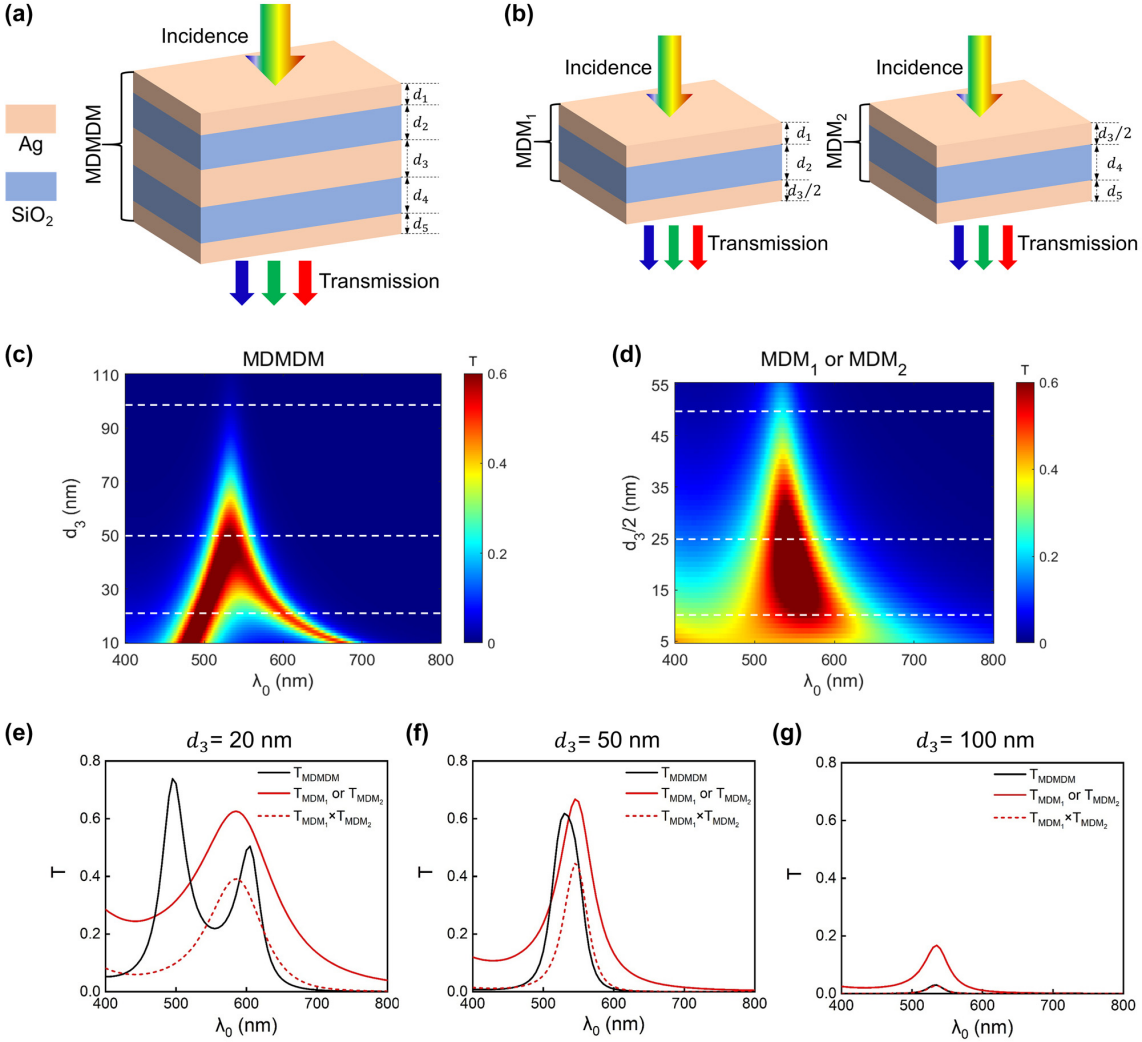
Comparison between the MDMDM coupled cavity structure and the MDM individual cavity structure. (a–b) Schematic diagrams of the proposed MDMDM structure (a), and the associated two stacked MDM structures, respectively denoted as MDM_1_ and MDM_2_ (b). (c–d) The contour plots of the simulated transmission spectrum from the MDMDM (c), and MDM (d) structures, as functions of the Ag layer thickness (*d*_3_ or *d*_3_/2) and illumination wavelength. In the simulation, the SiO_2_ layer thicknesses are fixed as 126 nm (*d*_2_ = *d*_4_ = 126 nm), and the two outermost Ag layer thicknesses are fixed as 20 nm (*d*_1_ = *d*_5_ = 20 nm). (e–g) Representative transmission spectra of MDMDM and MDM structures with three different *d*_3_ values (20 nm, 50 nm, and 100 nm). Here, the solid black curves represent the spectra of the MDMDM structure, the solid red curves represent the spectra of the MDM structure (the MDM_1_ and MDM_2_ structures exhibit identical transmission spectra), and the dashed red curves represent the multiplication of the spectra from the MDM_1_ and MDM_2_ structures.

Intuitively, one might expect the transmission spectrum of such MDMDM structure to be the multiplication of the spectra from two individual MDM FP cavities. However, we will show that with a carefully chosen layer configuration, the MDMDM structure instead displays a coupled resonance characteristic and consequently, exhibits a transmission spectrum of both large peak intensity and high spectral purity compared to that from an individual MDM FP structure. Figure 2a and b illustrate the schematic diagrams of the proposed MDMDM structure and the associated two stacked MDM structures (denoted as MDM_1_ and MDM_2_). For the ease of subsequent analysis, the fused silica substrate is neglected. It is worth noting that the conclusions derived from this simplified analysis also apply to cases where the fused silica substrate is present, and the associated details are elaborated in Supplementary Information, Section V.

The contour plots of the simulated transmission spectra from the MDMDM and MDM structures, as functions of middle Ag thickness (*d*_3_) and illumination wavelength, are displayed in Figure 2c and d, respectively. For both structures under numerical evaluation, the SiO_2_ layer thicknesses are set at 126 nm (*d*_2_ = *d*_4_ = 126 nm, corresponding to a typical green color device), and the thicknesses of the two outermost Ag layers are set at 20 nm (*d*_1_ = *d*_5_ = 20 nm). In addition, the middle Ag layer thickness (*d*_3_) of the MDMDM structure, which is twice the bottom (top) Ag layer thickness of the MDM_1_ (MDM_2_) structure, varies from 10 nm to 110 nm. It is evident that the two plots exhibit dramatically different characteristics. In the MDMDM case (Figure 2c), two resonant peaks initially exist when the middle Ag layer is thin (*d*_3_ < ∼40 nm). As *d*_3_ increases, the two peaks gradually approach each other and eventually merge into a single one when *d*_3_ > ∼40 nm. In contrast, the MDM structure always exhibits a single resonant peak whose central wavelength remains basically invariant as *d*_3_ increases, and the associated FWHM width gets narrower as the Ag layer thickness (*d*_3_/2) increases (Figure 2d). Such spectral narrowing behavior corresponds well to the typical characteristic of an FP cavity.

Representative transmission spectra of devices with three different *d*_3_ values (20 nm, 50 nm, and 100 nm) are displayed in Figure 2e–g. In these figures, the solid black curves represent the spectra of the MDMDM structure, the solid red curves represent those of the MDM structure (the MDM_1_ and MDM_2_ structures exhibit identical transmission spectra), and the dashed red curves represent the multiplication of the spectra from the two MDM structures. When *d*_3_ is thin (e.g., *d*_3_ = 20 nm), the two constituent cavities (MDM_1_ and MDM_2_) affect each other, and consequently, the MDMDM structure exhibits two discrete transmission resonances (Figure 2e). The induced resonance effect leads to a higher peak intensity compared to both the spectrum from the individual MDM structure and the multiplicated one of the two MDM structures. However, the color purity is compromised due to the discrete resonances centered at two different wavelengths.

When *d*_3_ gets too thick (e.g., *d*_3_ = 100 nm), the two constituent MDM cavities behave independently, and the induced resonance effect merely exits. Consequently, the transmission spectrum of the MDMDM structure is simply the multiplication of the spectra from the two cascaded MDM cavities (Figure 2g). In such case, the peak intensity of the MDMDM structure is significantly lower than that of the individual MDM structure, resulting in compromised color brightness.

When the middle Ag layer has a proper thickness (e.g., *d*_3_ = 50 nm), the two discrete resonances in the MDM cavities overlap spectrally, giving rise to a single resonance peak from the MDMDM structure (Figure 2f). We refer to the mode in such MDMDM structure as the coupled cavity resonance (CCR) mode. Facilitated by such coupled cavity resonance, the device’s transmission spectrum exhibits a peak intensity slightly lower than that of the spectrum from an individual MDM structure but significantly higher than the multiplicated spectrum of the two MDM structures. Simultaneously, such induced transmission only occurs within a narrow wavelength range, with an FWHM linewidth around 50 nm. Moreover, due to the relatively thick middle metallic layer, undesired sideband transmissions are effectively suppressed down to ∼0 %, further enhancing purity of the generated color. We also compare the spectra generated by the single cavity (MDM) and coupled cavity (MDMDM) in the CIE chromaticity diagram (Section VI, Supplementary Information). The color coordinate points associated with the coupled cavity structure are positioned much closer to the outer edge of the CIE diagram compared to the coordinate points associated with the single cavity structure, indicating the improved color purity generated by the MDMDM coupled cavity structures.

It is worthwhile to note that the thicknesses of the top and bottom Ag layers (*d*_1_ and *d*_5_) also impact the optimal choice of the middle Ag layer thickness (*d*_3_). For instance, when *d*_1_ and *d*_5_ are set at 10 nm, the optimal *d*_3_ value is 30 nm. Conversely, when *d*_1_ and *d*_5_ are 30 nm, the optimal *d*_3_ value increases to 70 nm. Additional details can be found in Section VII of the Supplementary Information. Choosing relatively thin metallic layers results in transmission spectra with high peak intensity but broader linewidth. On the other hand, selecting thicker metallic layers results in spectra with narrower linewidth but reduced peak intensity. Balancing color purity and brightness, we have chosen the combination of *d*_1_ = *d*_5_ = 20 nm and *d*_3_ = 50 nm in this study. Researchers are encouraged to choose values based on their specific design targets.

**Figure 3:**
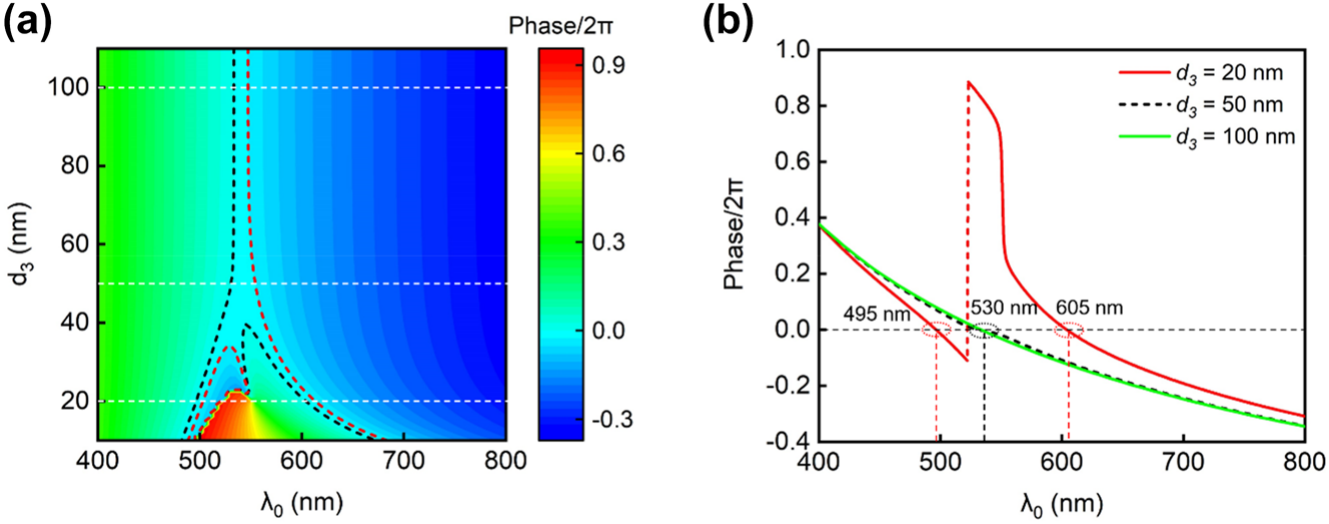
The net phase shift within an individual SiO2 cavity. (a) Calculated net phase shift inside the top SiO_2_ cavity as functions of the incident wavelength and the middle Ag layer thickness (*d*_3_). (b) Net phase shift profiles under three representative *d*_3_ values (20 nm, 50 nm, and 100 nm). The choice of thickness values for other constituent layers in the MDMDM structure is the same as the values in a typical green color device (listed in Table 1).

The trend of the two discrete resonances merging with the increase of the middle Ag layer thickness (*d*_3_) can be understood by examining the net phase shift (*δ*) inside an individual SiO_2_ cavity. The net phase shift includes the reflection phase shifts associated with the top and bottom Ag–SiO_2_ interfaces, along with the round-trip propagation phase shift inside the SiO_2_ cavity. Resonance occurs when the net phase shift equals multiples of 2π radians (*δ* = 2mπ, m = 0, ±1, ±2, ...), resulting in a transmission spectrum with enhanced intensity. Figure 3a depicts the calculated net phase shift inside the top SiO_2_ cavity of a green color device as functions of the incident wavelength and the middle Ag layer thickness (*d*_3_). In Figure 3a, the light blue region between the dashed black and red curves represents the spectral region where a peak transmission occurs (i.e., *δ* ≈ 0). When *d*_3_ is thin (*d*_3_ < ∼40 nm), there are two separated regions enclosed by the black and red curves. This corresponds to the observed fact that the MDMDM structure with a thin *d*_3_ layer will generate two transmission peaks. As *d*_3_ increases, the two initially-separated regions will gradually merge into a single one. This corresponds to the observed fact that the MDMDM structure will only generate one transmission peak when *d*_3_ > ∼40 nm.

Figure 3b illustrates three representative net phase shift profiles under different *d*_3_ values (20 nm, 50 nm, and 100 nm). When *d*_3_ = 20 nm, there are two wavelength points (*λ*_0_ = 495 nm and 605 nm) where the net phase shift equals 0. In contrast, for larger *d*_3_ values (*d*_3_ = 50 nm), there is only one wavelength point (*λ*_0_ = 530 nm) corresponding to the zero-value net phase shift, and the wavelength point stays invariant as *d*_3_ increases from 50 nm to 100 nm. Such observed trend of the two discrete resonances merging in the MDMDM structure, as revealed through the net phase shift analysis, closely aligns with the simulated transmission spectra shown in Figure 2.

**Figure 4:**
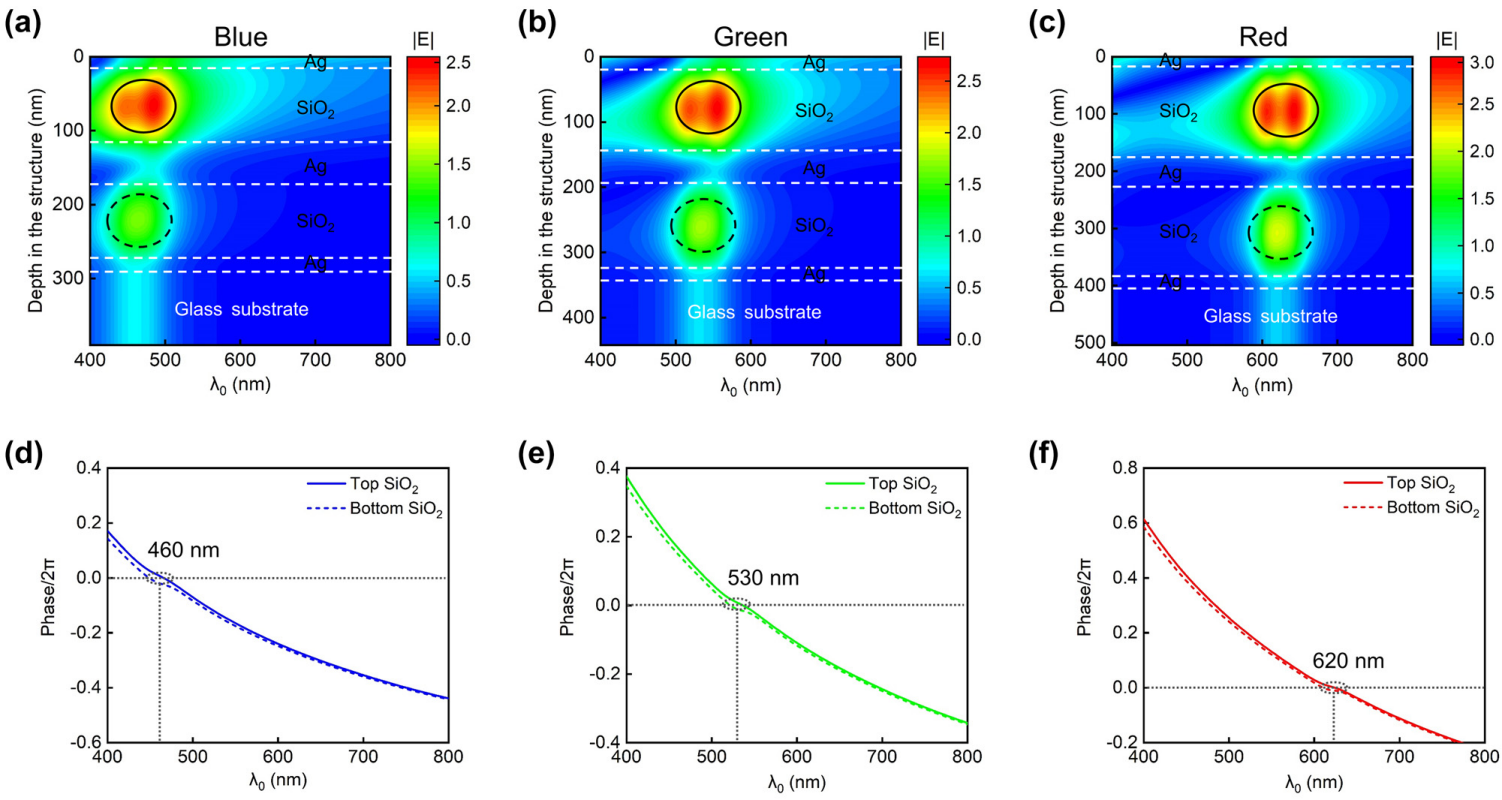
Electric field distribution and net phase shift analysis for RGB devices. (a–c) Wavelength-dependent electric field distributions for three devices corresponding to RGB color generation. Solid black circles and dashed black circles, respectively, denote the distinct resonances located in the top and bottom MDM cavities. (d–f) Calculated net phase shifts as a function of illumination wavelength inside the top and bottom SiO_2_ cavities for each device. For all devices, the two curves closely overlap across the whole visible range, exhibiting nearly identical resonant wavelengths.

To further elucidate the resonance behavior of the MDMDM structure, we conduct the electric field distribution analysis for three representative devices, each tailored for RGB color generation (see Table 1 for the devices’ structural parameters). Plane-wave illumination is incident from the air side. As shown in Figure 4a–c, each device manifests two distinct resonances within the top and bottom MDM cavities, spectrally overlapping with each other. The top SiO_2_ cavity, being closer to the incident light, exhibits a more pronounced resonance. The observed electric field distributions align with the calculated net phase shifts as a function of illumination wavelength inside the top and bottom SiO_2_ layers (Figure 4d–f). In these figures, solid curves represent the net phase shift inside the top SiO_2_ cavity, while dashed curves depict the net phase shifts inside the bottom SiO_2_ cavity. Resonance occurs when the net phase shift equals multiples of 2*π* radians. Remarkably, for all three devices, the two curves closely overlap across the whole visible range, exhibiting nearly identical resonant wavelengths (where the net phase shift is zero). Such spectral overlap contributes to the narrow and strong transmission peaks observed in the MDMDM structures.

We also perform additional analysis of the transmission spectra from triple-cavity and four-cavity structures (details listed in Section VIII, Supplementary Information). The analysis shows that employing more cavities does not significantly improve the linewidth of the transmission spectrum, while at the same time, compromises the peak intensity. Therefore, choosing a dual-cavity structure not only ensures both color purity and brightness but also maintains a straightforward and easy-to-implement configuration.

**Figure 5:**
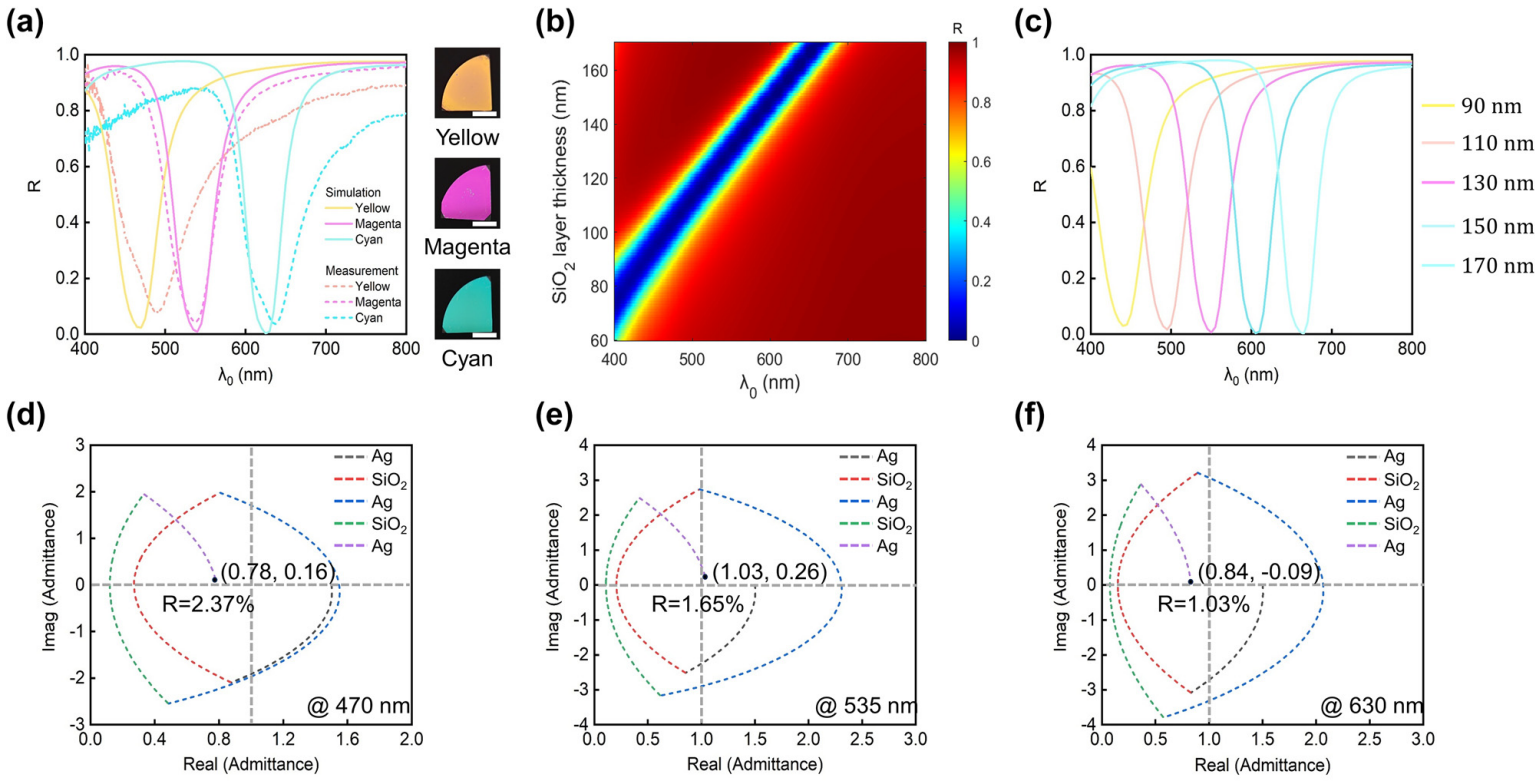
Reflection-type structural color generation based on the Ag-SiO_2_-Ag-SiO_2_-Ag configuration. (a) Left panel: simulated (solid curves) and measured (dashed curves) reflection spectra for cyan (C), magenta (M), and yellow (Y) devices. Right panel: optical images (reflection-view) of the fabricated samples. Scale bars: 1.0 cm. (b) Contour plot of the simulated reflection spectrum as functions of the SiO_2_ layer thickness and illumination wavelength. Here, *d*_1_ = *d*_5_ = 20 nm and *d*_3_ = 50 nm. (c) Five representative reflection spectra with SiO_2_ layer thicknesses ranging from 90 nm to 170 nm in 20 nm increments. (d–f) Optical admittance diagrams for the dip wavelength of the reflection spectrum from each device. Reflectivity from the device is related to the distance from the final admittance point to the point of air (1.0, 0).

In addition to the RGB colors observed in transmission view, the devices also display complementary CMY colors (cyan, magenta, and yellow) in their reflection view. In Figure 5a, the solid curves represent simulated reflection spectra under normal incidence for the three devices listed in Table 1. Each device generates cyan (C), magenta (M), and yellow (Y) colors, with resonant wavelengths at 630 nm, 535 nm, and 470 nm, respectively. Their reflection spectra exhibit dips down to 0 % and sideband reflection intensity up to 100 %, complementing the transmission spectra. The dashed curves in Figure 5a depict the measured reflection spectra of the fabricated samples, closely aligning with the simulated ones. Reflection-view optical images of the fabricated samples are displayed in the right panel of Figure 5a.

Similar to the case of transmission-type color generation, the dip position of the device’s reflection spectrum can be readily adjusted by varying the thickness of the SiO_2_ layers while fixing those of the Ag layers. The reflection dip shifts from approximately 400 nm to 655 nm as the thickness of the two SiO_2_ layers varies from 60 nm to 170 nm (Figure 5b), with the thickness of the top and bottom Ag layers fixed at 20 nm (*d*_1_ = *d*_5_ = 20 nm) and the middle Ag layer at 50 nm (*d*_3_ = 50 nm). Figure 5c displays five representative reflection spectra, with SiO_2_ layer thickness ranging from 90 nm to 170 nm with a 20 nm increment. All spectra exhibit close-to-zero resonant dip values and near-unity sideband reflection intensities.

To better understand the working mechanism of the reflection-type structural colors generated by the MDMDM structures, we employ optical admittance diagram analysis (Figure 5d–f). Three wavelengths of 470 nm, 535 nm, and 630 nm, respectively corresponding to the reflection dip position in the spectrum of each device, are selected for study. Optical admittance (Y) is the inverse of impedance and is calculated as $Y=\sqrt{\varepsilon /\mu }$ , where *ε* and *μ* represent the relative permittivity and permeability of a material, respectively. Optical admittance often equates to a material’s complex refractive index, considering the relative permeability of common materials is one at optical frequencies. In our analysis, the admittance begins from the fused silica substrate and follows a circular or spiral trajectory based on the optical constants (*n*, *κ*) and film thickness of each layer. Reflectivity (*R*) of the device is related to the distance between the terminating admittance point of the MDMDM structure and the admittance point of air (1, 0):

(1) $$R={\left(\frac{1-\left(x+iy\right)}{1+\left(x+iy\right)}\right)}^{2}$$

Here, *x* and *y* denote the real and imaginary components of the final admittance point, respectively. The calculated final admittance points for the three devices are (0.78, 0.16), (1.03, 0.26), and (0.84, −0.09), respectively. These coordinate points are all located close to the admittance point of air, indicating a low reflection intensity. Such analysis aligns with the observed reflection spectra, where dip intensities are significantly suppressed to approximately 0 % (2.37 %, 1.65 %, and 1.03 %, respectively, for the yellow, magenta, and cyan devices).

**Figure 6:**
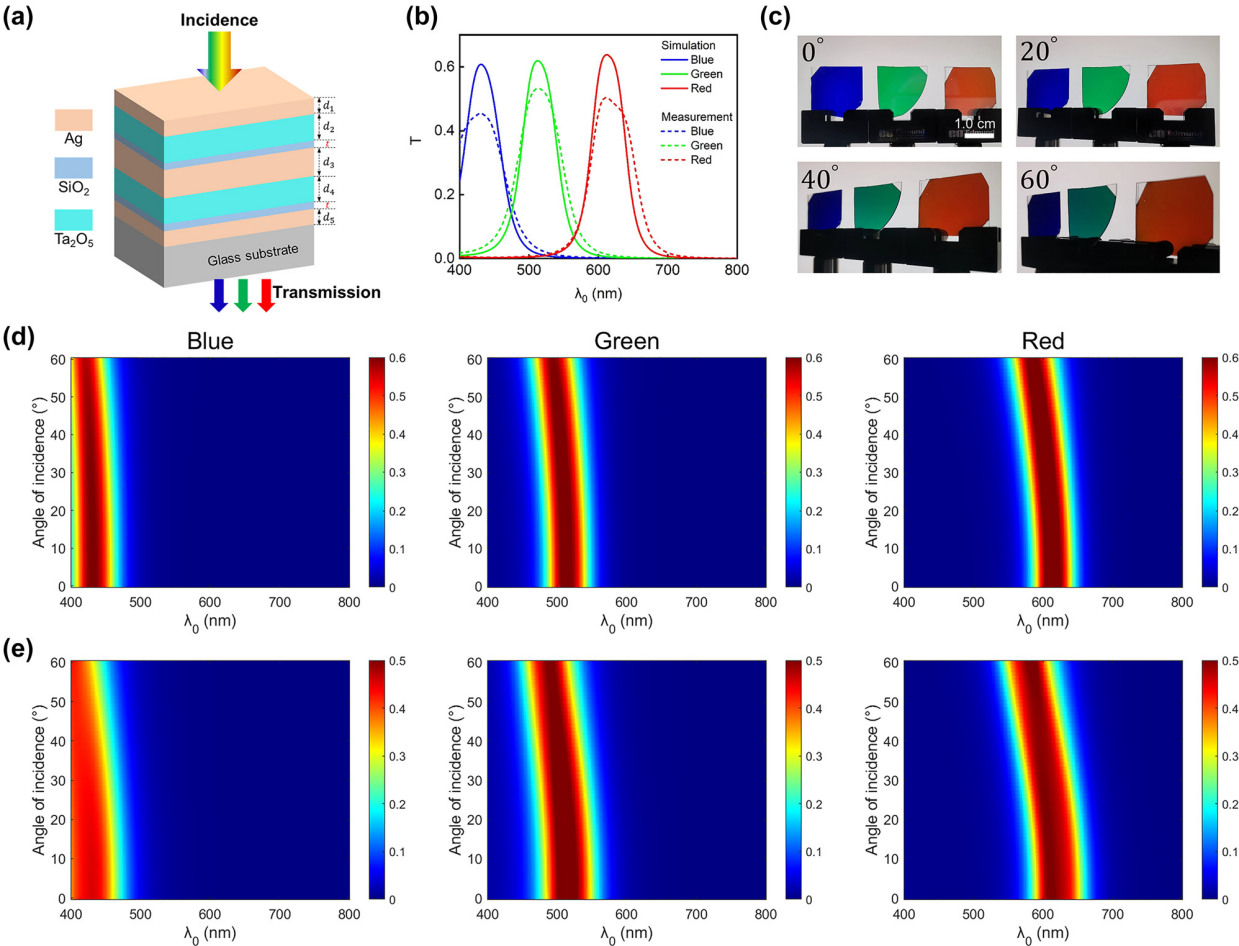
Angle-robust transmission-type structural color generation based on the Ag-Ta_2_O_5_-Ag- Ta_2_O_5_-Ag configuration. (a) Schematic diagram of the Ta_2_O_5_-based MDMDM structure. (b) Simulated (solid curves) and measured (dashed curves) transmission spectra of Ta_2_O_5_-based devices for blue (B), green (G), and red (R) colors under normal incidence. (c) Optical images (transmission-view) of the fabricated RGB samples taken at four different viewing angles (0°, 20°, 40°, 60°). Scale bar: 1.0 cm. (d–e) Simulated (d) and measured (e) angle-resolved transmission spectra under unpolarized illumination light.

Notably, the proposed MDMDM structure can employ an array of transparent dielectric materials in the visible [46], [47], [48], [49], [50], including aluminum oxide (Al_2_O_3_), hafnium dioxide (HfO_2_), tantalum pentoxide (Ta_2_O_5_), titanium dioxide (TiO_2_), silicon nitride (SiN_*x*_), and others. Specifically, the use of a dielectric with high refractive index enhances the device’s angle-robust response (Detailed explanation can be found in Section IX, Supplementary Information). As a proof of concept, we present another group of devices incorporating Ta_2_O_5_ as the dielectric layers (Figure 6a). The Ta_2_O_5_ is deposited through a reactive electron beam deposition process using oxygen (O_2_) gas. The measured refractive index and extinction coefficient of deposited Ta_2_O_5_ layer are plotted in Figure S1 of Supplementary Information, exhibiting a high refractive index (*n* ∼ 2.2 at 532 nm) and zero extinction coefficient across the whole visible spectrum. To safeguard the Ag layer from potential degradation during the fabrication process, a 10-nm-thick (*t* = 10 nm) SiO_2_ layer is deposited onto the Ag layer as a protection layer. Detailed fabrication process is elaborated in the Methods Section. For RGB devices, the thicknesses of the top, middle, and bottom Ag layers are all set to be 20 nm, 50 nm, and 20 nm, respectively. The thicknesses of the two Ta_2_O_5_ layers are kept identical and set to be 86 nm (red device), 62 nm (green device), and 42 nm (blue device). Detailed structural configurations for RGB color generation as well as the associated peak transmission wavelengths are listed in Section X, Supplementary Information. The solid curves in Figure 6b depict the simulated transmission spectra under normal light illumination for three representative devices, exhibiting peak transmission wavelengths of 612 nm (corresponding to red color generation), 512 nm (green color generation), and 430 nm (blue color generation). The dashed curves in Figure 6b depict the measured transmission spectra, exhibiting a reasonable correspondence with the simulated ones.

To illustrate the angularly-insensitive response of the Ta_2_O_5_-based MDMDM structures, we present both the calculated and measured transmission spectra of the RGB devices (Figure 6d and e). Unpolarized illumination light is used for all cases. The devices exhibit nearly invariant transmission spectra as the angle of incidence increases from 0° to 60°. Photographs of the fabricated devices taken at four different viewing angles (namely, 0°, 20°, 40°, and 60°) are displayed in Figure 6c, revealing distinct RGB colors that remain consistent appearance across the viewing angles.

**Figure 7:**
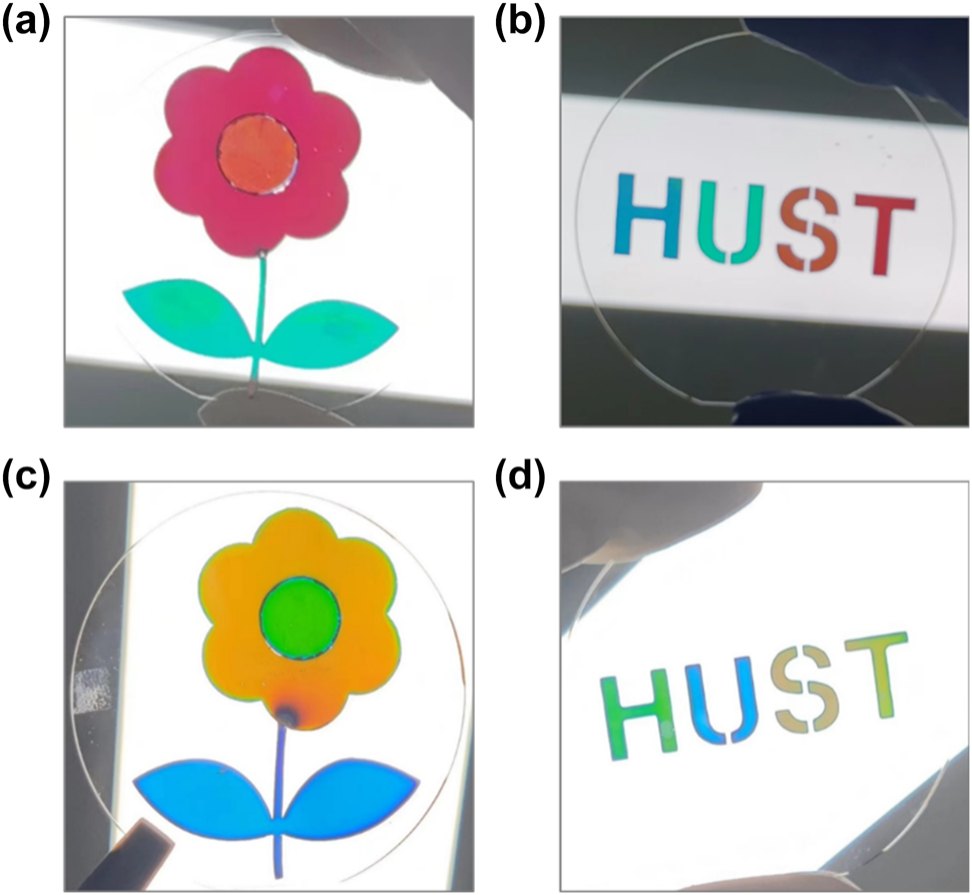
Color image patterns on 2-inch fused silica substrates. (a) Photo of a colored flower based on the Ag–SiO_2_–Ag–SiO_2_–Ag configuration. The SiO_2_ layer thicknesses for the areas of the green branches and leaves, the yellow center, and the red petals are 120 nm, 155 nm, and 175 nm, respectively. (b) Photo of colored “HUST” letters based on the Ag–SiO_2_–Ag–SiO_2_–Ag configuration. The SiO_2_ layer thicknesses for the areas of “H”, “U”, “S”, and “T” are 112 nm, 120 nm, 155 nm, and 175 nm, respectively. (c) Photo of a colored flower based on the Ag–Ta_2_O_5_–Ag–Ta_2_O_5_–Ag configuration. The Ta_2_O_5_ layer thicknesses for the areas of the blue branches and leaves, the green center, and the yellow petals are 57 nm, 80 nm, and 63 nm, respectively. (d) Photo of colored “HUST” letters based on the Ag–Ta_2_O_5_–Ag–Ta_2_O_5_–Ag configuration. The Ta_2_O_5_ layer thicknesses for the areas of “H”, “U”, “S”, and “T” are 63 nm, 57 nm, 80 nm, and 75 nm, respectively. For all samples, the thicknesses of the top and bottom Ag layers are fixed at 20 nm, and the middle Ag layer is 50 nm.

Finally, it is worth noting that the proposed design can be employed to generate color images over a single substrate using an array of fabrication techniques such as shadow mask method [51], [52], direct-write grayscale lithography [53], grayscale stencil lithography [54], and nanoimprint lithography [55]. As a proof of concept, we employ the shadow mask method to fabricate a series of color image patterns on 2-inch fused silica substrates, using both the Ag–SiO_2_–Ag–SiO_2_–Ag and Ag–Ta_2_O_5_–Ag–Ta_2_O_5_–Ag configurations discussed previously (Figure 7).

## 3 Conclusions

In conclusion, our study introduces a novel approach based on the metal/dielectric/metal/dielectric/metal (MDMDM) configuration for creating large-scale structural colors characterized by exceptional purity and brightness. Leveraging the coupled cavity resonance between adjacent FP cavities, our MDMDM design yields sharp and intense transmission peaks with significantly suppressed sideband intensities. Unlike previous attempts based on multilayered thin films, our approach uses only a single combination of one type of dielectric and one type of metal, without requiring any anti-reflection layers or changing layer choices for different colors. Our experimental results demonstrate the successful realization of high-performance transmission-type red, green, and blue colors using an Ag/SiO_2_/Ag/SiO_2_/Ag configuration. The measured transmission spectra exhibit narrow resonant linewidths (∼60 nm), high peak efficiencies (>40 %), and effectively suppressed sideband intensities (∼0 %). By adjusting the thickness of SiO_2_ layers, we showcase the versatility of our approach in producing a wide range of structural colors spanning the entire visible spectrum, surpassing the color gamut of standard color spaces. Additionally, our MDMDM structure exhibits high-performance reflection-type complementary colors, further expanding its potential applications. More importantly, the proposed design is compatible with various choices of metallic and dielectric layers and can be utilized to create different colors over a single substrate. For instance, we demonstrate angle-robust structural colors using high-index Ta_2_O_5_ as the dielectric layer, and fabricate a series of color image patterns on 2-inch fused silica substrates using the shadow mask method. Furthermore, by incorporating materials with tunable thickness (e.g., polyvinyl alcohol hydrogel [56], [57]) or refractive index (e.g., liquid crystal [58], epsilon-near-zero material [59], and electrochemical material [60]), our design can facilitate dynamically tunable color generation. Our work offers a novel route for realizing large-scale and high-performance structural colors in a cost-effective manner, and could find potential applications in various areas such as advanced printing and decoration, optical anti-counterfeiting, and colored photovoltaics.

## 4 Methods

### 4.1 Device fabrication

The devices are fabricated using an electron beam evaporation system at room temperature with a base pressure of around 6.0 × 10^−4^ Pa. The deposition rates for Ag and SiO_2_ layers are 3 Å s^−1^ and 2 Å s^−1^, respectively. The Ta_2_O_5_ film is deposited at a rate of 2 Å s^−1^ with an O_2_ gas flow rate of 15 standard cubic centimeters per minute (sccm). When fabricating the Ag/Ta_2_O_5_/Ag/Ta_2_O_5_/Ag based devices, a 10-nm-thick SiO_2_ layer is first deposited onto the Ag layer as a protection layer to prevent potential degradation during the fabrication process.

### 4.2 Refractive index characterization

The refractive indices (*n*) and extinction coefficients (*κ*) of the deposited thin films are characterized by a reflection-mode spectroscopic ellipsometer using the interference enhancement method [61]. This characterization is performed at three different angles of incidence (55°, 65°, and 75°) with respect to the normal to the plane of the layer under study.

### 4.3 Device simulation and characterization

Transfer matrix method (TMM) is used to calculate the transmission spectra, reflection spectra, net phase shifts, and electric field distributions of the proposed devices. In calculating the phase shifts associated with the round-trip propagation as well as interface reflection, the angle function of MATLAB is employed, which restricts the obtained phase shift values to the range of –*π* to *π*. The transmission and reflection spectra under unpolarized illumination are obtained by averaging the device’s spectra under TE and TM polarized illuminations. The measured transmission and reflection spectra under normal incidence, as well as the angle-resolved transmission spectra are all obtained using a spectroscopic ellipsometer.

## Supplementary Material

Supplementary Material Details

## References

[1] Wang D. Y., Liu Z. Y., Wang H. Z., Li M. X., Guo L. J., Zhang C. (2023). Structural color generation: from layered thin films to optical metasurfaces. *Nanophotonics*.

[2] Xuan Z. Y., Li J. Y., Liu Q. Q., Yi F., Wang S. W., Lu W. (2021). Artificial structural colors and applications. *Innovation*.

[3] Ji C. G., Lee K. T., Xu T., Zhou J., Park H. J., Guo L. J. (2017). Engineering light at the nanoscale: structural color filters and broadband perfect absorbers. *Adv. Opt. Mater.*.

[4] Kristensen A. (2017). Plasmonic colour generation. *Nat. Rev. Mater.*.

[5] Song M. (2019). Colors with plasmonic nanostructures: a full-spectrum review. *Appl. Phys. Rev.*.

[6] Lee T., Jang J., Jeong H., Rho J. (2018). Plasmonic- and dielectric-based structural coloring: from fundamentals to practical applications. *Nano Converg.*.

[7] Hail C. U., Schnoering G., Damak M., Poulikakos D., Eghlidi H. (2020). A plasmonic painter’s method of color mixing for a continuous red-green-blue palette. *ACS Nano*.

[8] Liu Y. J. (2019). Structural color three-dimensional printing by shrinking photonic crystals. *Nat. Commun.*.

[9] Dai C. J. (2023). Direct-printing hydrogel-based platform for humidity-driven dynamic full-color printing and holography. *Adv. Funct. Mater.*.

[10] Zhu J. (2020). Three-dimensional cavity-coupled metamaterials for plasmonic color and real-time colorimetric biosensors. *Nanoscale*.

[11] Balaur E. (2021). Colorimetric histology using plasmonically active microscope slides. *Nature*.

[12] Ji C. G., Zhang Z., Masuda T., Kudo Y., Guo L. J. (2019). Vivid-colored silicon solar panels with high efficiency and non-iridescent appearance. *Nanoscale Horiz.*.

[13] Kim Y., Son J., Shafian S., Kim K., Hyun J. K. (2018). Semitransparent blue, green, and red organic solar cells using color filtering electrodes. *Adv. Opt. Mater.*.

[14] Hong W., Yuan Z. K., Chen X. D. (2020). Structural color materials for optical anticounterfeiting. *Small*.

[15] Jung C. (2021). Near-zero reflection of all-dielectric structural coloration enabling polarization-sensitive optical encryption with enhanced switchability. *Nanophotonics*.

[16] Cheben P., Halir R., Schmid J. H., Atwater H. A., Smith D. R. (2018). Subwavelength integrated photonics. *Nature*.

[17] Banerjee P. P. (2024). Prediction of metallo-dielectric transmission filter performance based on underlying dispersion relations. *J. Opt. Soc. Am. B*.

[18] Al-Ghezi H., Gnawali R., Banerjee P. P., Sun L., Slagle J., Evans D. (2020). 2 x 2 anisotropic transfer matrix approach for optical propagation in uniaxial transmission filter structures. *Opt. Express*.

[19] Uddin M. J., Magnusson R. (2013). Highly efficient color filter array using resonant Si_3_N_4_ gratings. *Opt. Express*.

[20] Koirala I., Shrestha V. R., Park C. S., Lee S. S., Choi D. Y. (2017). Polarization-controlled broad color palette based on an ultrathin one-dimensional resonant grating structure. *Sci. Rep.*.

[21] Kaplan A. F., Xu T., Guo L. J. (2011). High efficiency resonance-based spectrum filters with tunable transmission bandwidth fabricated using nanoimprint lithography. *Appl. Phys. Lett.*.

[22] Keshavarz Hedayati M., Elbahri M. (2017). Review of metasurface plasmonic structural color. *Plasmonics*.

[23] Song M. W. (2023). Versatile full-colour nanopainting enabled by a pixelated plasmonic metasurface. *Nat. Nanotechnol.*.

[24] Wu Y.-K. R., Hollowell A. E., Zhang C., Guo L. J. (2013). Angle-insensitive structural colours based on metallic nanocavities and coloured pixels beyond the diffraction limit. *Sci. Rep.*.

[25] Baek K., Kim Y., Mohd-Noor S., Hyun J. K. (2020). Mie resonant structural colors. *ACS Appl. Mater. Interfaces*.

[26] Wu Y., Chen Y., Song Q., Xiao S. (2021). Dynamic structural colors based on all-dielectric Mie resonators. *Adv. Opt. Mater.*.

[27] Jang J., Badloe T., Yang Y., Lee T., Mun J., Rho J. (2020). Spectral modulation through the hybridization of mie-scatterers and quasi-guided mode resonances: realizing full and gradients of structural color. *ACS Nano*.

[28] Zhao J. (2019). Defining deep-subwavelength-resolution, wide-color-gamut, and large-viewing-angle flexible subtractive colors with an ultrathin asymmetric Fabry–Perot lossy cavity. *Adv. Opt. Mater.*.

[29] Kats M. A., Blanchard R., Genevet P., Capasso F. (2013). Nanometre optical coatings based on strong interference effects in highly absorbing media. *Nat. Mater.*.

[30] Liu K., Lin Z., Han B., Hong M., Cao T. (2024). Non-volatile dynamically switchable color display via chalcogenide stepwise cavity resonators. *Opto-Electron. Adv.*.

[31] Li Z. Y., Butun S., Aydin K. (2015). Large-area, lithography-free super absorbers and color filters at visible frequencies using ultrathin metallic films. *ACS Photonics*.

[32] Kim D. (2022). Manipulation of resonance orders and absorbing materials for structural colors in transmission with improved color purity. *Opt. Express*.

[33] Ji C. G., Lee K. T., Guo L. J. (2019). High-color-purity, angle-invariant, and bidirectional structural colors based on higher-order resonances. *Opt. Lett.*.

[34] Lee K. T., Han S. Y., Li Z. J., Baac H. W., Park H. J. (2019). Flexible high-color-purity structural color filters based on a higher-order optical resonance suppression. *Sci. Rep.*.

[35] Yang Z. M. (2016). Reflective color filters and monolithic color printing based on asymmetric Fabry-Perot cavities using nickel as a broadband absorber. *Adv. Opt. Mater.*.

[36] Yang Z. M., Ji C. G., Liu D., Guo L. J. (2019). Enhancing the purity of reflective structural colors with ultrathin bilayer media as effective ideal absorbers. *Adv. Opt. Mater.*.

[37] Liu J. T., Feng K., Wang Y. S., Li Q. Y., Chen N., Bu Y. K. (2022). High-color-purity, high-brightness and angle-insensitive red structural color. *Chin. Opt. Lett.*.

[38] Yang Z. M., Ji C. G., Cui Q. Y., Guo L. J. (2020). High-purity hybrid structural colors by enhancing optical absorption of organic dyes in resonant cavity. *Adv. Opt. Mater.*.

[39] Lee K. T., Han S. Y., Park H. J. (2017). Omnidirectional flexible transmissive structural colors with high-color-purity and high-efficiency exploiting multicavity resonances. *Adv. Opt. Mater.*.

[40] Zhang C., Ji C. G., Park Y.-B., Guo L. J. (2021). Thin-metal-film-based transparent conductors: material preparation, optical design, and device applications. *Adv. Opt. Mater.*.

[41] Zhang C. (2017). High-performance doped silver films: overcoming fundamental material limits for nanophotonic applications. *Adv. Mater.*.

[42] McPeak K. M. (2015). Plasmonic films can easily be better: rules and recipes. *ACS Photonics*.

[43] Yang W. H. (2020). All-dielectric metasurface for high-performance structural color. *Nat. Commun.*.

[44] Yang B. (2019). Ultrahighly saturated structural colors enhanced by multipolar-modulated metasurfaces. *Nano Lett.*.

[45] ElKabbash M. (2023). Fano resonant optical coatings platform for full gamut and high purity structural colors. *Nat. Commun.*.

[46] Zhang C. (2020). Low-loss metasurface optics down to the deep ultraviolet region. *Light Sci. Appl.*.

[47] Zhang C. (2024). Tantalum pentoxide: a new material platform for high-performance dielectric metasurface optics in the ultraviolet and visible region. *Light Sci. Appl.*.

[48] Huo P. (2020). Photonic spin-multiplexing metasurface for switchable spiral phase contrast imaging. *Nano Lett.*.

[49] Colburn S. (2018). Broadband transparent and CMOS-compatible flat optics with silicon nitride metasurfaces. *Opt. Mater. Express*.

[50] Yu Z. (2024). Genetic algorithm assisted meta-atom design for high-performance metasurface optics. *Opto-Electron. Sci.*.

[51] Lee J. Y., Lee K.-T., Seo S., Guo L. J. (2014). Decorative power generating panels creating angle insensitive transmissive color. *Sci. Rep.*.

[52] Yan Z. (2021). Floating solid-state thin films with dynamic structural colour. *Nat. Nanotechnol.*.

[53] Yang Z. M. (2017). Microscopic interference full-color printing using grayscale-patterned Fabry-Perot resonance cavities. *Adv. Opt. Mater.*.

[54] Li X., Tan Z. J., Fang N. X. (2020). Grayscale stencil lithography for patterning multispectral color filters. *Optica*.

[55] Baek S. (2020). Solution-processable multi-color printing using UV nanoimprint lithography. *Nanotechnology*.

[56] Zhang J. (2021). Grayscale-patterned metal-hydrogel-metal microscavity for dynamic multi-color display. *Nanophotonics*.

[57] Wang Z. (2022). Real-time tunable nanoprinting-multiplexing with simultaneous meta-holography displays by stepwise nanocavities. *Adv. Funct. Mater.*.

[58] Franklin D. (2015). Polarization-independent actively tunable colour generation on imprinted plasmonic surfaces. *Nat. Commun.*.

[59] Mirshafieyan S. S., Gregory D. A. (2018). Electrically tunable perfect light absorbers as color filters and modulators. *Sci. Rep.*.

[60] Wang Z. (2020). Towards full-colour tunability of inorganic electrochromic devices using ultracompact Fabry-Perot nanocavities. *Nat. Commun.*.

[61] Zhang C. (2018). Robust extraction of hyperbolic metamaterial permittivity using total internal reflection ellipsometry. *ACS Photonics*.

