# Supplementary material for: Large-scale high purity and brightness structural color generation in layered thin film structures via coupled cavity resonance
Source: Nanophotonics. 2024 Oct 30;13(24):4491–503. doi: 10.1515/nanoph-2024-0471 (PMC11636523; doi:10.1515/nanoph-2024-0471)
Supplement: Supplementary file 1 — Supplementary Material Details [file j_nanoph-2024-0471_suppl_001.pdf]

Supplementary information for

Large-scale High Purity and Brightness Structural Color Generation in Layered Thin Film Structures via Coupled Cavity Resonance

Danyan Wang[1+], Chengang Ji[2+ *], Moxin Li[1], Zhenyu Xing[1], Hao Gao[1], Xiaochan Li[2], Huixian Zhou[1], Yuhui Hu[1], Zhelin Lin[1], and Cheng Zhang[1*]

[1] School of Optical and Electronic Information & Wuhan National Laboratory for Optoelectronics, Huazhong University of Science and Technology, Wuhan, Hubei 430074, China

[2] Ningbo Inlight Technology Co., Ltd, Ningbo, Zhejiang 315500, China

[+] Equal contributors

[*] Corresponding authors. Chengang Ji, ji@inlighttec.com; Cheng Zhang, cheng.zhang@hust.edu.cn

This Supplementary Information includes:

Section I. Optical properties of the deposited Ag, SiO_2, and Ta_2O_5 thin films

Section II. Transmittance of the fused silica substrate over the visible range

Section III. Angular responses of the SiO_2-based devices under TE- and TM-polarized illumination

Section IV. Color coordinate calculation in the CIE 1931 XYZ chromaticity diagram

Section V. Comparison of the transmission spectra of layered thin film structures with and without the fused silica substrate

Section VI. Color coordinate comparison between MDM and MDMDM structures in CIE 1931 XYZ color psace

Section VII. Influence of top and bottom Ag layer thicknesses on the optimal choice of the middle Ag layer thickness

Section VIII. Transmission spectra of the dual-cavity, triple-cavity, and four-cavity structures for blue, green, and red color generation.

Section IX. Discussion of the device's angle-robust performance when using high-refractive-index dielectric material

Section X. Geometric parameters of Ta_2O_5-based devices for angle-robust RGB color generation

I. Optical properties of the deposited Ag, SiO_2, and Ta_2O_5 thin films

A 20-nm-thick Ag film is deposited using an electron beam evaporation system onto a silicon wafer with a 300-nm-thick thermal oxide layer. The film's refractive index (n) and extinction coefficient (κ) are characterized by a reflection-mode spectroscopic ellipsometer using the interference enhancement method. As displayed in Fig. S1a, the 20-nm-thick Ag film exhibits a high extinction coefficient (κ) but near-zero refractive index (n) over the entire visible band (400-800 nm). Such near-zero n leads to low absorption from the Ag film, as described by the following equation, which is used to model the light absorption of a planar thin film.

$$A(x,\lambda) = \frac{2\pi c \varepsilon_0 n\kappa}{\lambda} |E(x,\lambda)|^2 \quad (1)$$

where c is the speed of light in free space, ε_0 is the permittivity of free space, $|E(z,\lambda)|^2$ is the electric field intensity of wavelength λ at position x within the thin film, and n and κ respectively denote the refractive index and extinction coefficient of a thin film.

A 100-nm-thick SiO_2 film is deposited using an electron beam evaporation system on a silicon wafer. The film's refractive index (n) and extinction coefficient (κ) are characterized by a reflection-mode spectroscopic ellipsometer. A refractive index of 1.5 (n = 1.5) with zero extinction coefficient (κ = 0) is achieved, as displayed in Fig. S1b.

A 100-nm-thick Ta_2O_5 film is deposited using an electron beam evaporation system on a silicon wafer with a ~300-nm-thick thermal oxide layer. The film's refractive index (n) and extinction coefficient (κ) are characterized by a reflection-mode spectroscopic ellipsometer. As displayed in Fig. S1c, the film shows a high refractive index (n > 2.1) and zero extinction coefficient (κ=0) over the whole visible region.

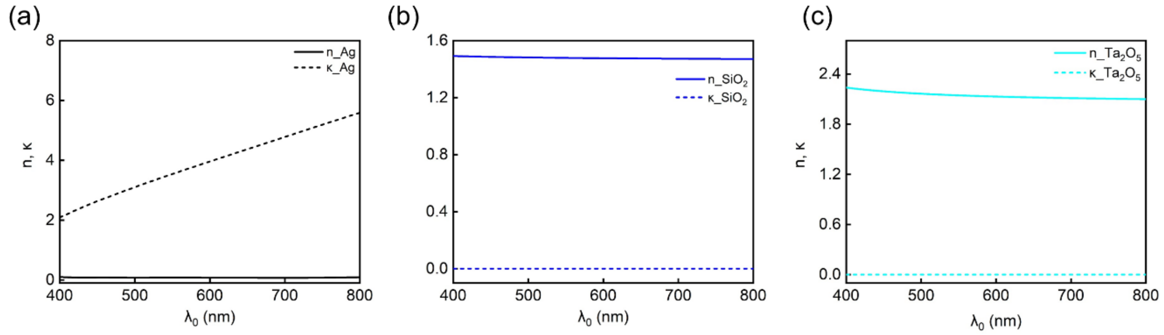

Figure S1. Measured refractive indices and extinction coefficients of a 20-nm-thick Ag film (a), a 100-nm-thick SiO_2 film (b), and a 100-nm-thick Ta_2O_5 film (c).

II. Transmittance of the fused silica substrate over the visible range

As displayed in Fig. S2, the 0.5-mm-thick fused silica wafer provides a high optical transmittance (> 92%) over the entire optional bandwidth in this study (400-800 nm).

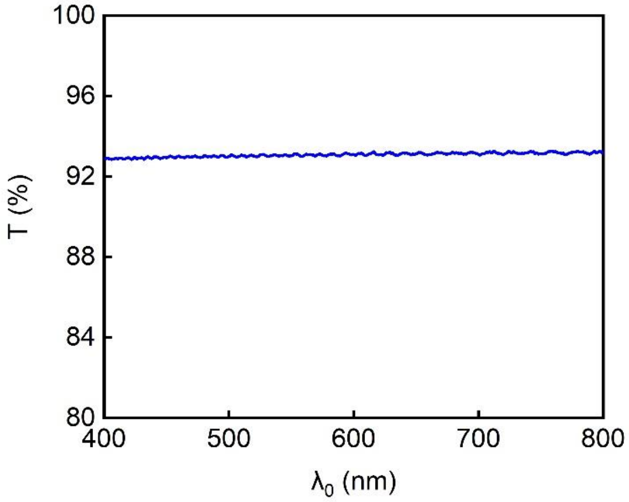

Figure S2. Measured transmittance versus wavelength of a 0.5-mm-thick fused silica wafer over the visible range.

III. Angular responses of the SiO_2-based devices under TE- and TM-polarized illumination

Figures S3 and S4 display the simulated and measured angle-resolved transmission spectra of the SiO_2-based devices under TE- and TM-polarized illuminations, respectively. The devices' peak wavelengths shift toward the blue region as the angle of incidence increases from 0° to 60°, showing the angle-sensitive characteristic of the SiO_2-based configuration.

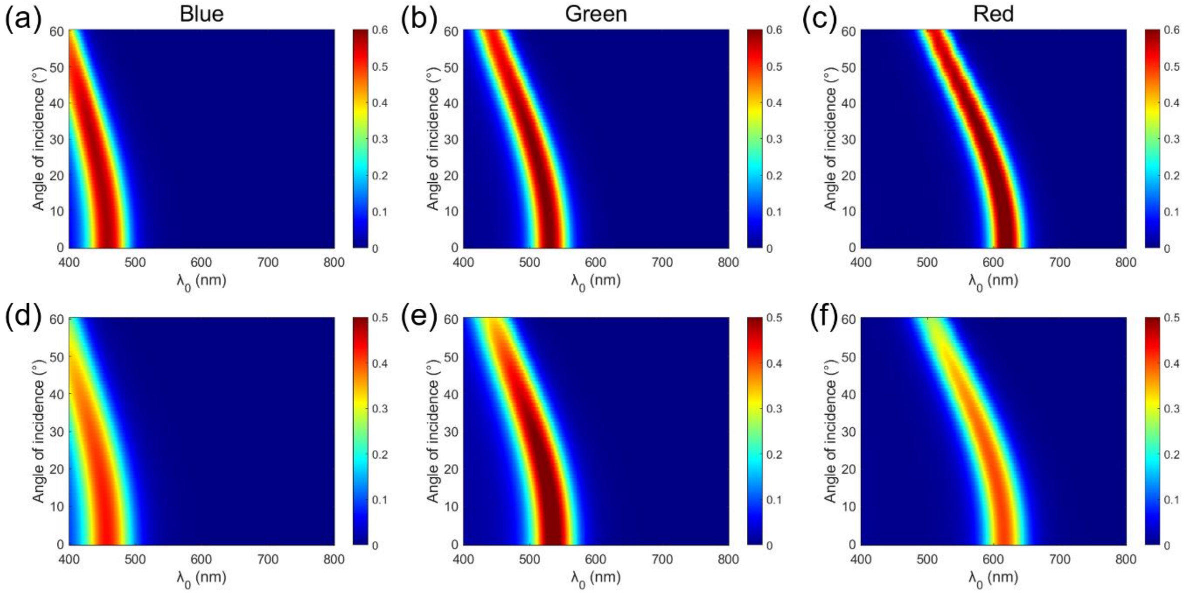

Figure S3. Angular responses of the SiO_2-based devices under TE-polarized illumination. (a-c) Simulated and (d-f) measured angle-resolved transmission spectra of the SiO_2-based devices under TE-polarized incident light. Here, TE-polarization refers to the electric field perpendicular to the plane of incidence.

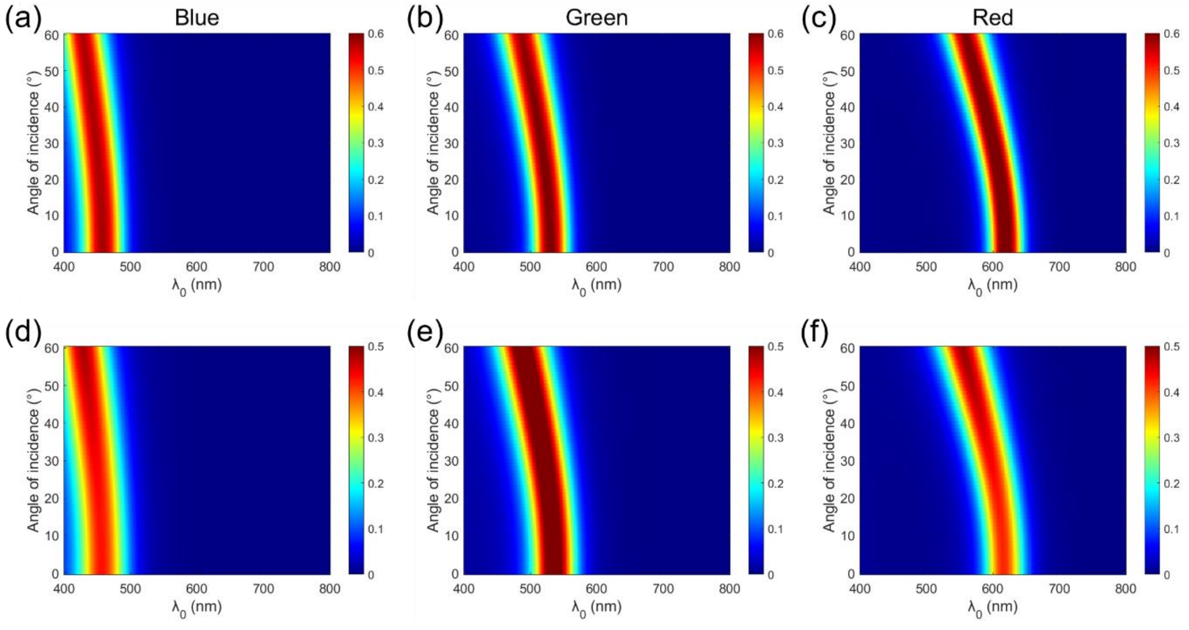

Figure S4. Angular responses of the SiO_2-based devices under TM-polarized illumination. (a-c) Simulated and (d-f) measured angle-resolved transmission spectra of the SiO_2-based devices under TM-polarized incident light. Here, TM-polarization refers to the electric field parallel to the plane of incidence.

IV. Color coordinate calculation in the CIE 1931 XYZ chromaticity diagram

The CIE 1931 XYZ chromaticity diagram (Fig. S5a) is created as a perceptual color map to contrast different color stimuli. Each point in the diagram denotes a specific color, and the associated coordinate (x, y) can be obtained by calculating the following equations (Eq. 1):

$$\begin{cases} x = \frac{X}{X+Y+Z} \\ y = \frac{Y}{X+Y+Z} \end{cases} \tag{1}$$

Where X, Y, and Z represent the tristimulus values, which can be obtained by solving the corresponding integral equations (Eq. 2):

$$\begin{cases} X = \frac{1}{k}\int_{\lambda_1}^{\lambda_2} \bar{x}(\lambda)I(\lambda)T(\lambda)d\lambda \\ Y = \frac{1}{k}\int_{\lambda_1}^{\lambda_2} \bar{y}(\lambda)I(\lambda)T(\lambda)d\lambda \\ Z = \frac{1}{k}\int_{\lambda_1}^{\lambda_2} z(\lambda)I(\lambda)T(\lambda)d\lambda \end{cases} \tag{2}$$

Here, $[\lambda_{1,}\ \lambda_2]$ represents the spectrum range, and is set to be [400 nm, 800 nm] in our study. Additionally, $\bar{x}(\lambda), \bar{y}(\lambda),$ and $\bar{z}(\lambda)$ represent the color matching functions, and are shown in Fig. S5b. $\mathrm{I}(\lambda)$ denotes the relative spectral power distribution of the illumination light source. Standard illuminant E, an equal-energy radiator with a color temperature of approximately 5455 K, is selected as the light source in our work. $\mathrm{T}(\lambda)$ represents the simulated or measured transmission spectrum in our work. *k* is a normalized factor and is defined as:

$$k = \int_{\lambda_1}^{\lambda_2} \bar{y}(\lambda)I(\lambda)d\lambda \tag{3}$$

In the CIE 1931 XYZ chromaticity diagram, the color gradually transitions from blue to green and then to red when moving the coordinate point clockwise from the bottom-left corner to the top-left corner and then to the bottom-right corner. Coordinates closer to the outer edge of the diagram correspond to colors of high purity, while points closer to the center (white point) correspond to colors of low purity.

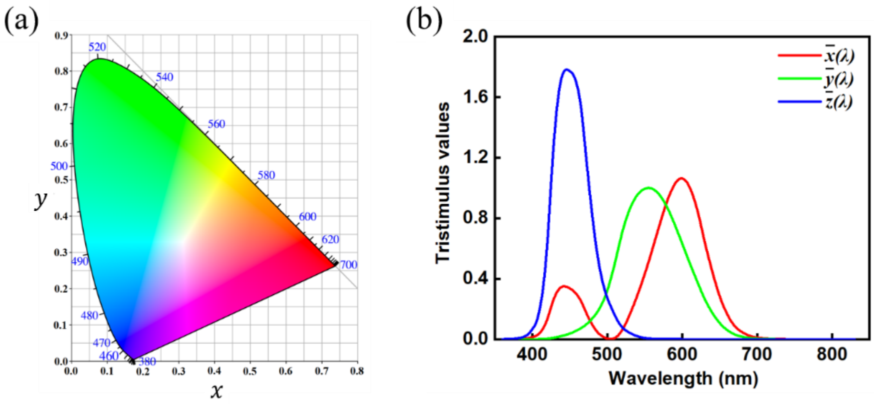

Figure S5. (a) The CIE 1931 XYZ chromaticity diagram. (b) Color matching functions $\bar{x}(\lambda), \bar{y}(\lambda), and\ \bar{z}(\lambda)$.

V. Comparison of the transmission spectra of layered thin film structures with and without the fused silica substrate

The schematic diagrams of the proposed MDMDM structure and the associated two stacked MDM structures (MDM_1 and MDM_2) are displayed in Figs. S6a and S6b, respectively. These diagrams include the fused silica substrate. Using a green color device as an example, the transmission spectra of layered thin film structures with (solid curves) and without (dashed curves) the fused silica substrate are calculated. As displayed in Fig. S7, the spectrum profiles calculated with a silica substrate nearly overlap with the spectral profiles without a silica substrate.

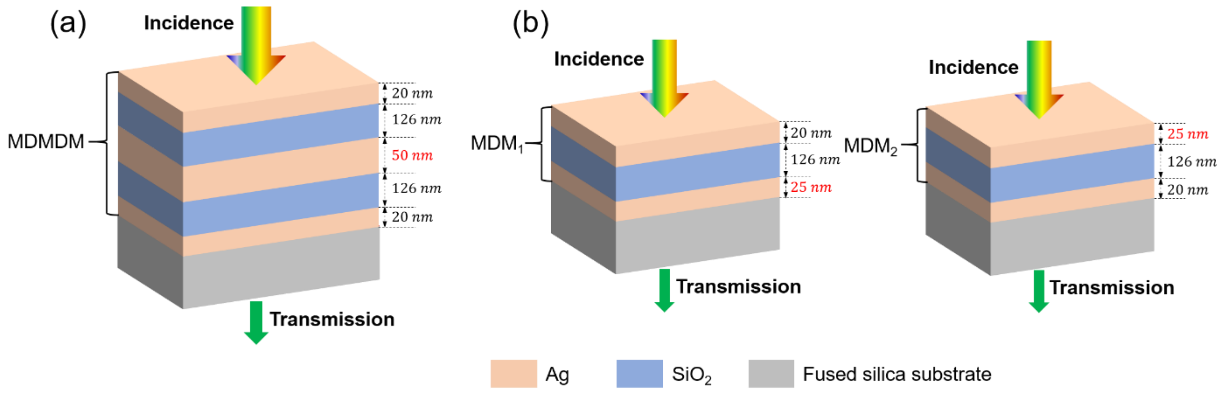

Figure S6. Schematic diagrams of the proposed MDMDM structure (a), and the associated two stacked MDM structures denoted as MDM_1 and MDM_2 (b).

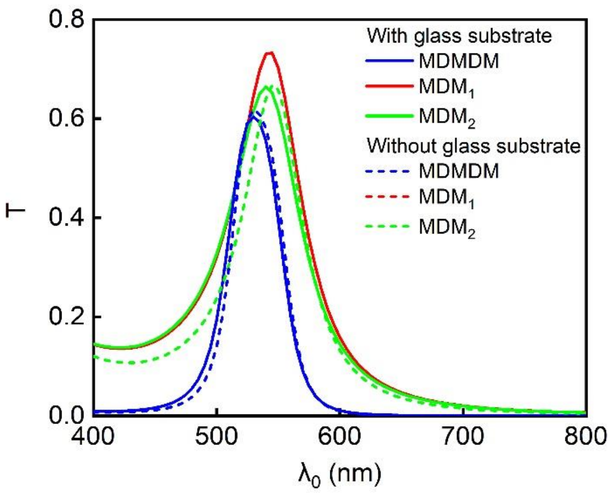

Figure S7. Transmission spectra of the MDMDM, MDM_1, and MDM_2 structures with (solid curves) and without (dashed curves) the fused silica substrate under normal incidence.

VI. Color coordinate comparison between MDM and MDMDM structures in CIE 1931 XYZ color psace

As displayed in Fig. S8, the color coordinate points associated with the coupled cavity (MDMDM) are positioned much closer to the outer edge of the CIE chromaticity diagram compared to the coordinate points associated with the single cavity (MDM), indicating the improved color purity generated by the MDMDM coupled cavity structures.

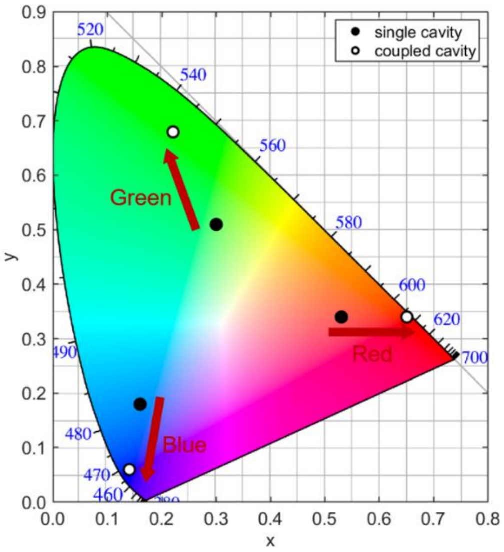

Figure S8. The color coordinate points of the MDM-based single cavity (blue (0.16, 0.18), green (0.30, 0.51), and red (0.53, 0.34)) and the MDMDM-based coupled cavity (blue (0.14, 0.06), green (0.22, 0.68), and red (0.65, 0.34)) in the CIE 1931 XYZ chromaticity diagram.

VII. Influence of top and bottom Ag layer thicknesses on the optimal choice of the middle Ag layer thickness

Figures. S9a and S9b respectively display the contour plots of the simulated transmission spectrum of the MDMDM structure under two sets of top and bottom Ag layer thicknesses ($d_1 = d_5 = 10\ nm,$ and $d_1 = d_5 = 30\ nm$). For both structures under numerical evaluation, the SiO_2 layer thicknesses are set at 126 nm ($d_2 = d_4 = 126\ nm$), and the middle Ag layer thickness (d_3) varies from 10 nm to 110 nm. In the case of a 10-nm-thick top and bottom Ag layer ($d_1 = d_5 = 10\ nm$), two resonant peaks initially exist when the middle Ag layer thickness is thin (d_3< ~ 20 nm, Fig. S9a). As d_3 increases, the two peaks gradually approach each other and eventually merge into a single one when d_3 > ~ 20 nm. In contrast, the MDMDM structure with a 30-nm-thick top and bottom Ag layer ($d_1 = d_5 = 30\ nm$) exhibits a single resonant peak when d_3 > ~ 60 nm (Fig. S9b). In Fig. S9c, two representative transmission spectra of the devices are plotted under specific conditions. As displayed in the plot, using relatively thin metallic layers (i.e., $d_1 = d_5 = 10\ nm$, and $d_3 = 30\ nm$) leads to a transmission spectrum with a high peak intensity but broader linewidth (with FWHM of approximately 130 nm). Conversely, using thicker metallic layers (i.e., $d_1 = d_5 = 30\ nm$, and $d_3 = 70\ nm$) results in a transmission spectrum with a narrower linewidth but reduced peak intensity (with a peak transmission of approximately 0.14).

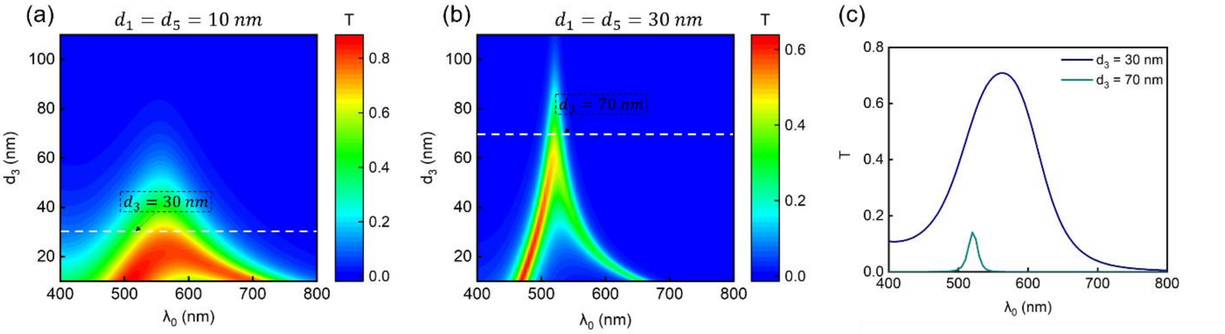

Figure S9. (a, b) 2D plots of the calculated transmission spectra of the MDMDM cavity as a function of the middle Ag layer thickness (d_3) and incident wavelength. In the case of Fig. S9a, the top and bottom Ag layer is chosen to be 10 nm in thickness; while in the case of Fig. S9b, the top and bottom Ag layer is chosen to be 30 nm in thickness. In all cases, the two SiO_2 layers are chosen to be 126 nm in thickness. (c) Calculated transmission spectra under two different d_3 values (30 nm and 70 nm).

VIII. Transmission spectra of the dual-cavity, triple-cavity, and four-cavity structures for blue, green, and red color generation.

Figure S10 displays the schematics of the dual-cavity, triple-cavity, and four-cavity structures for blue-, green-, and red-color generation, along with their simulated transmission spectra. It can be seen that employing more cavities does not significantly improve the linewidth of the transmission spectrum, while simultaneously compromising the peak intensity of the transmission spectrum (details listed in Table S1). Therefore, choosing a dual-cavity structure not only ensures both color purity and brightness but also maintains a straightforward and easy-to-implement configuration.

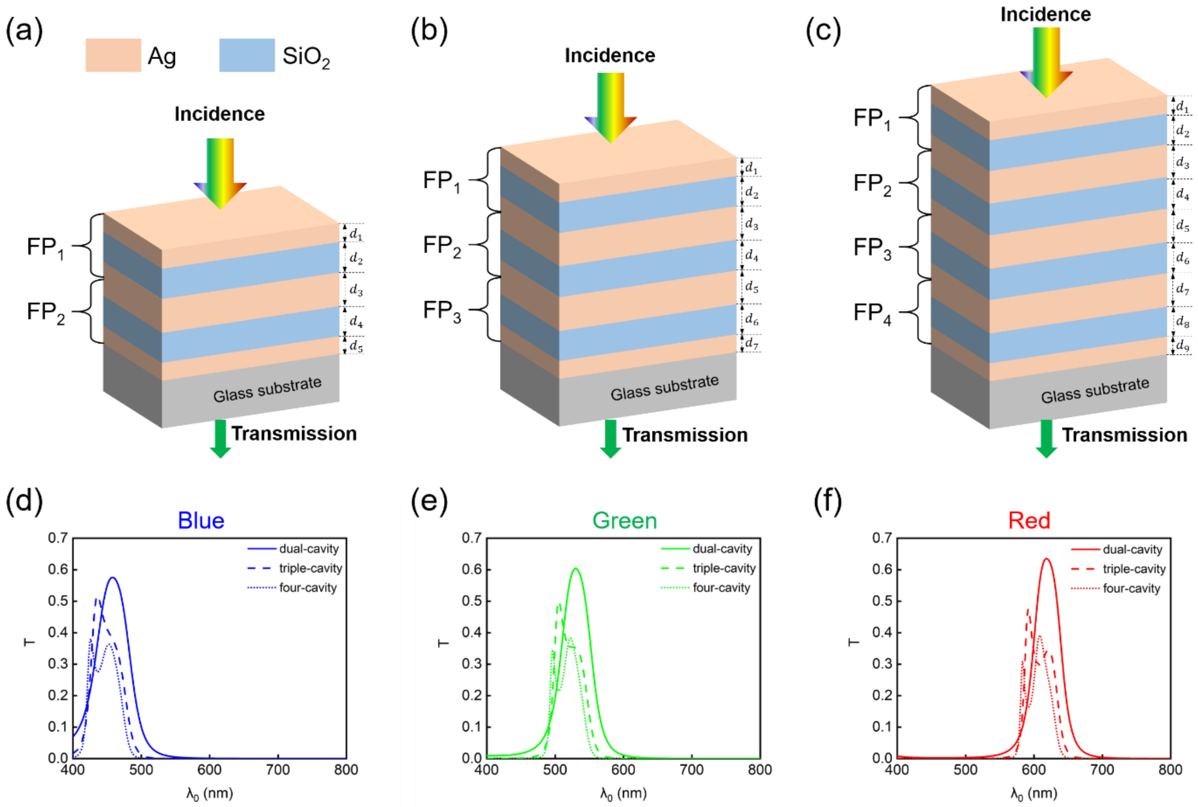

Figure S10. Transmission spectra of the dual-cavity, triple-cavity, and four-cavity structures for blue, green, and red color generation. (a-c) Schematic diagrams of the dual-cavity, triple-cavity, and four-cavity configurations. (d-f) Simulated transmission spectra of these structures for blue, green, and red colors. The detailed geometric parameters are listed in Table S1.

Table S1. Geometric parameters of the dual-cavity, triple-cavity, and four-cavity devices for blue, green, and red color generation, and the associated linewidth and peak intensity of their simulated transmission spectra.

Colors	Configurations	Geometry parameters	FWHM	Peak transmission
Blue	dual-cavity	$d_1 = d_5 = 20\,nm;\ d_3 = 50\,nm$<br>$d_2 = d_4 = 100\,nm$	55 nm	0.58
	triple-cavity	$d_1 = d_7 = 20\,nm;\ d_3 = d_5 = 50\,nm$<br>$d_2 = d_4 = d_6 = 100\,nm$	48 nm	0.52
	four-cavity	$d_1 = d_9 = 20\,nm;\ d_3 = d_5 = d_7 = 50\,nm$<br>$d_2 = d_4 = d_6 = d_8 = 100\,nm$	51 nm	0.38
Green	dual-cavity	$d_1 = d_5 = 20\,nm;\ d_3 = 50\,nm$<br>$d_2 = d_4 = 126\,nm$	48 nm	0.6
	triple-cavity	$d_1 = d_7 = 20\,nm;\ d_3 = d_5 = 50\,nm$<br>$d_2 = d_4 = d_6 = 126\,nm$	45 nm	0.5
	four-cavity	$d_1 = d_9 = 20\,nm;\ d_3 = d_5 = d_7 = 50\,nm$<br>$d_2 = d_4 = d_6 = d_8 = 126\,nm$	46 nm	0.38
Red	dual-cavity	$d_1 = d_5 = 20\,nm;\ d_3 = 50\,nm$<br>$d_2 = d_4 = 157\,nm$	45 nm	0.63
	triple-cavity	$d_1 = d_7 = 20\,nm;\ d_3 = d_5 = 50\,nm$<br>$d_2 = d_4 = d_6 = 157\,nm$	47 nm	0.47
	four-cavity	$d_1 = d_9 = 20\,nm;\ d_3 = d_5 = d_7 = 50\,nm$<br>$d_2 = d_4 = d_6 = d_8 = 157\,nm$	31 nm	0.4

IX. Discussion of the device's angle-robust performance when using high-refractive-index dielectric material

The angle-robust performance facilitated by a high-refractive-index dielectric can be intuitively attributed to the fact that a high-refractive-index medium reduces the refraction angle when light enters the structure according to the Snell's Law.

The angle-robust performance can also be explained using a standard Fabry-Pérot (FP) cavity model, where a transparent dielectric layer is sandwiched between two identical metallic mirrors (Fig. S11). The transmittance of the FP cavity can be expressed as:

$$T_{FP} = \frac{(1-R)^2}{1+R^2-2Rcos\delta} \tag{4}$$

where R is the reflection coefficient of the metal film. δ is the total phase shift accumulated during a single round trip within the cavity and can be expressed as:

$$\delta = (\frac{2\pi}{\lambda}) \cdot \mathrm{n} \cdot 2\mathrm{d} \cdot \cos\theta_2 \tag{5}$$

Here, θ_2 represents the angle of refraction at the metal/dielectric interface. Transmission maxima occur at certain wavelengths when the total phase shift δ is an integral number of 2π radians (i.e., $\delta = 2\mathrm{m}\pi$).

According to the Snell's Law ($\sin\theta_1 = nsin\theta_2$), the FP cavity's resonance wavelength λ_r can be expressed as:

$$\lambda_r = 2d\sqrt{n^2 - sin^2\theta_1} \tag{6}$$

The variation in the resonant wavelength as a function of angle of incidence can be expressed as:

$$\left|\frac{\Delta\lambda_r}{\Delta\theta_1}\right| \approx \frac{2d \cdot sin\theta_1 \cdot cos\theta_1}{\sqrt{n^2 sin^2\theta_1}} \tag{7}$$

According to Equation (7), the angular sensitivity of the FP cavity is inversely proportional to the refractive index of the dielectric layer. Therefore, using high-refractive-index dielectric materials enhances the device's angle-robust performance.

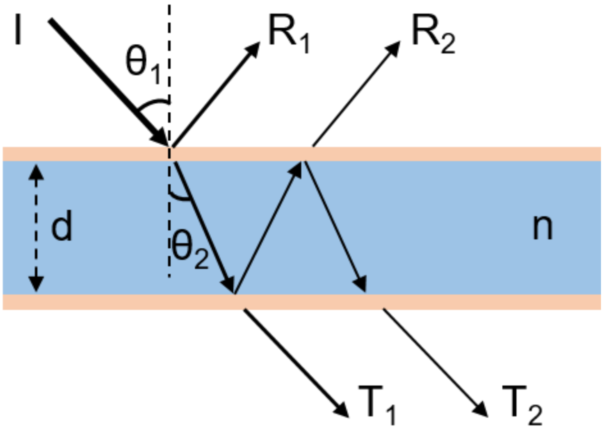

Figure S11. Schematic diagram of a typical FP cavity model, consisting of a transparent dielectric layer sandwiched between two identical metallic mirrors. Here, n and d respectively represent the refractive index and thickness of the dielectric layer. I is the incidence, and R and T denote the reflection and transmission. θ_1 and θ_2 represent angles of incidence and refraction at the metal/dielectric interface on the input side, respectively.

X. Geometric parameters of Ta_2O_5-based devices for angle-robust RGB color generation

Table S2 lists the detailed structural configurations for RGB color generation as well as the associated peak transmission wavelengths.

Table S2. Geometric parameters of Ta_2O_5-based MDMDM structures for angle-robust RGB color generation

Color	Peak λ (nm)	d_1 (nm)	d_2 (nm)	d_3 (nm)	d_4 (nm)	d_5 (nm)	t (nm)
Red	612	20	86	50	86	20	10
Green	512	20	62	50	62	20	10
Blue	430	20	42	50	42	20	10
